# Nestin expression is upregulated in the fibrotic rat heart and is localized in collagen-expressing mesenchymal cells and interstitial CD31^(+)^- cells

**DOI:** 10.1371/journal.pone.0176147

**Published:** 2017-04-27

**Authors:** Vanessa Hertig, Kim Tardif, Marc Andre Meus, Natacha Duquette, Louis Villeneuve, Fanny Toussaint, Jonathan Ledoux, Angelino Calderone

**Affiliations:** 1 Research Center, Montreal Heart Institute and Université de Montréal, Montréal, Québec, Canada; 2 Department of Pharmacology & Physiology, Université de Montréal, Québec, Montréal, Canada; 3 Department of Medicine, Université de Montréal, Québec, Montréal, Canada; Albert Einstein College of Medicine, UNITED STATES

## Abstract

Renal and lung fibrosis was characterized by the accumulation of collagen-immunoreactive mesenchymal cells expressing the intermediate filament protein nestin. The present study tested the hypothesis that nestin expression was increased in the hypertrophied/fibrotic left ventricle of suprarenal abdominal aorta constricted adult male Sprague-Dawley rats and induced in ventricular fibroblasts by pro-fibrotic peptide growth factors. Nestin protein levels were upregulated in the pressure-overloaded left ventricle and expression positively correlated with the rise of mean arterial pressure. In sham and pressure-overloaded hearts, nestin immunoreactivity was detected in collagen type I^(+)^-and CD31^(+)^-cells identified in the interstitium and perivascular region whereas staining was absent in smooth muscle α-actin^(+)^-cells. A significantly greater number of collagen type I^(+)^-cells co-expressing nestin was identified in the left ventricle of pressure-overloaded rats. Moreover, an accumulation of nestin^(+)^-cells lacking collagen, CD31 and smooth muscle α-actin staining was selectively observed at the adventitial region of predominantly large calibre blood vessels in the hypertrophied/fibrotic left ventricle. Angiotensin II and TGF-β_1_ stimulation of ventricular fibroblasts increased nestin protein levels via phosphatidylinositol 3-kinase- and protein kinase C/SMAD3-dependent pathways, respectively. CD31/eNOS^(+)^-rat cardiac microvascular endothelial cells synthesized/secreted collagen type I, expressed prolyl 4-hydroxylase and TGF-β_1_ induced nestin expression. The selective accumulation of adventitial nestin^(+)^-cells highlighted a novel feature of large vessel remodelling in the pressure-overloaded heart and increased appearance of collagen type I/nestin^(+)^-cells may reflect an activated phenotype of ventricular fibroblasts. CD31/collagen/nestin^(+)^-interstitial cells could represent displaced endothelial cells displaying an unmasked mesenchymal phenotype, albeit contribution to the reactive fibrotic response of the pressure-overloaded heart remains unknown.

## Introduction

Reactive fibrosis characterized by the uncontrolled synthesis and deposition of extracellular matrix proteins by ventricular fibroblasts represents a secondary pathological consequence of concentric cardiac hypertrophy.[1] The increased accumulation of interstitial collagen in the hypertrophied ventricle reduced ventricular compliance, compromised excitation-contraction coupling and represents an additional risk factor for the development of cardiac arrhythmias.[1,2] During embryogenesis, epithelial- and endothelial-mesenchymal transformation gave rise to adult ventricular fibroblasts.[3–5] Following the superimposition of a hemodynamic pressure-overload on the adult heart, the ensuing reactive fibrotic response was attributed primarily to the proliferation of resident ventricular fibroblasts.[3–5] It was generally acknowledged that during reactive and reparative fibrosis, normal fibroblasts acquired an activated myofibroblast phenotype characterized by smooth muscle α-actin expression, a greater proliferative response and synthesized higher levels of collagen and pro-fibrotic/pro-angiogenic peptide growth factors.[1–3, 6–8] However, the relative importance of smooth muscle α-actin remains a point of contention as depletion failed to inhibit wound healing and expression was not required in the transition of normal fibroblasts to a myofibroblast phenotype.[9,10] Moreover, smooth muscle α-actin was not detected in the preponderance of collagen type I-expressing mesenchymal cells identified in fibrotic lungs secondary to hypobaric hypoxia and the fibrotic heart following pressure-overload.[3,11] Work from our lab and others have reported that reactive and reparative fibrosis was characterized by the induction of the class VI intermediate filament protein nestin in a subpopulation of mesenchymal cells.[6,7,11–13] It was further revealed that following renal injury, the magnitude of the reactive fibrotic response positively correlated with the density of the nestin^(+)^-interstitial cells and exposure of renal-derived collagen-expressing fibroblasts to pro-fibrotic peptide growth factors increased nestin protein levels.[13] Biologically, several distinct functions were attributed to nestin including cellular proliferation, migration and a pro-survival anti-apoptotic phenotype.[6,7,14–17] Collectively, these observations provided the impetus to test the hypothesis that nestin protein levels were upregulated in the hypertrophied/fibrotic left ventricle following suprarenal abdominal aorta constriction of adult male Sprague-Dawley rats and induction of the intermediate filament protein in ventricular fibroblasts by putative pro-fibrotic peptide growth factors may represent a phenotypic marker of an activated state during reactive fibrosis.

## Materials and methods

### Ethics approval

The use and care of laboratory rodents was according to the Canadian Council for Animal Care and approved by the Animal Care Committee of the Montreal Heart Institute.

### Rat model of concentric hypertrophy, cardiac morphology and fibrosis

Suprarenal abdominal aorta constriction was performed on adult male Sprague-Dawley rats (9-11 weeks old; Charles Rivers, Montreal, Canada), employing a 21-gauge needle as previously described.[14] Prior to surgery, rats received a subcutaneous injection of buprenorphine (0.05 mg/kg; 6–8 hours prior to surgery) and subsequently anesthetized with 5% isoflurane and reduced to 1,5% during the surgical procedure. Following surgery, rats received two injections of buprenorphine at 6 hour intervals and a single dose of ketoprofen (5 mg/kg). Sham rats underwent the identical surgical procedure and analgesic treatment post-op. Two weeks following surgery, sham and banded rats were anesthetized with ketamine/xylazine (100/10 mg/kg) and mean arterial pressure and left ventricular function were determined with a Millar catheter, as previously described.[14] Following hemodynamic measurements, the heart was excised, the atria and ventricles weighed, immersed in liquid nitrogen and stored at -80°C. In a subpopulation of normal and suprarenal abdominal aorta constricted rats, the heart was excised and immediately fixed in formalin. Formalin-fixed tissue sections of 5–10 μm in thickness were stained with haematoxylin-phloxin-saffron (HPS) and Masson's trichrome to assess cardiac morphology and fibrosis respectively, as previously described.[14] Images were captured with the Olympus QICAM colour video camera interfaced with an Olympus CKX41 microscope and the program Image-Pro calculated collagen content and the data normalized to the area.

### Isolation and culturing of adult and neonatal rat ventricular fibroblasts

Adult and neonatal rat ventricular fibroblasts were isolated and cultured as previously described. [6,7] Experiments were performed on 1^st^ and 2^nd^ passaged fibroblasts plated at a density of 150–200 cells/mm^2^. Ventricular fibroblasts were plated in DMEM-low glucose (Hyclone laboratories, Logan, UT) supplemented with 7% heat-inactivated FBS and 1% penicillin-streptomycin for 36–48 hours, subsequently washed and maintained for 36–48 hours in DMEM-low glucose media supplemented with insulin (5 μg/ml), transferrin (5 μg/ml), and selenium (5 ng/ml) (BD bioscience, Bedford, MA) prior to the experimental protocol. Normal and pressure-overload adult ventricular fibroblasts were treated for 24 hrs with angiotensin II (1 μM; Sigma-Aldrich, St. Louis, MO), transforming growth factor-β_1_ (5 ng/ml; PeproTech, Rocky Hill, NJ) or epidermal growth factor (10 ng/ml; PeproTech). In neonatal ventricular fibroblasts, the PI3-K inhibitor LY294002 (10 μM; Enzo Life Sciences, Farmingdale, NY), the PKC inhibitor GF109203X (1 μM; Sigma-Aldrich) or the SMAD3 inhibitor SIS3 (5 μM; Sigma-Aldrich) was added 15–30 minutes prior to the treatment with angiotensin II (1 μM), transforming growth factor-β_1_ (5 ng/ml), or phorbol 12,13-dibutyrate (10^−7^ M; Sigma-Aldrich) for 24 hrs. In separate experiments, the cell permeable N-myristolated PKC (20–28) inhibitor (10 μM; Sigma-Aldrich) that selectively targeted the PKCα/β-isoforms was added for 24 hrs. ^3^H-thymidine uptake was measured as previously described.[6,7,14]

### Rat microvascular endothelial cells and human coronary artery endothelial cells

Primary rat cardiac microvascular endothelial cells (RMVECs) were purchased from Cell Biologics (Chicago IL). Primary RMVECs were initially plated in T25 flasks in complete rat endothelial cell media (Cell Biologics) supplemented with FBS to obtain a final concentration of 5% and grown until confluent. Thereafter, RMVECs were plated in T75 flasks, permitted to reach confluency, passaged 1:3 ratio and experiments were performed on passage 5–6. RMVECs were plated at a density of 10–20 cells/mm^2^ for 24–36 hrs in the rat endothelial cell media-5% FBS. Thereafter, RMVECs were washed and maintained in DMEM-low glucose media supplemented with insulin (5 μg/ml), transferrin (5 μg/ml), and selenium (5 ng/ml) for 36 hrs prior to experiment. RMVECs were subsequently treated for 24 or 48 hours with transforming growth factor-β_1_ (5 ng/ml; PeproTech, Rocky Hill, NJ) or epidermal growth factor (10 ng/ml; PeproTech). In a subset of immunofluorescence experiments, RMVECs were grown to confluency in rat endothelial cell media-5% FBS and subsequently fixed.

Human primary coronary artery endothelial cells (HCAEC; Lonza, Walkersville, MD) obtained from 2 distinct lots (#000419199, #0000303981) were initially plated (50 cells/mm^2^) in plasma-treated T75 flasks (Corning, NY) and cultured in media recommended by manufacturer (EGM^™^-2MV bullet kit, Lonza). Upon near confluency (~90%), cells were passaged in T75 flasks. At passage 6–9, coronary endothelial cells were plated in 12-well plates coated with 0.5% gelatin (Sigma-Aldrich, St. Louis, MO), grown in EGM^™^-2MV media and 24 hours later fixed for immunofluorescence.

### Western blot

Lysates (30–50 μg) prepared from the left ventricle of normal and suprarenal aorta constricted rats, adult and neonatal ventricular fibroblasts and rat microvascular endothelial cells were subjected to SDS-polyacrylamide gel (15%) electrophoresis and transferred to a PVDF membrane (Perkin Elmer Life Sciences, Boston, MA).[6,14] Antibodies used include mouse monoclonal anti-nestin (~240 KDa;1:500; EMD Millipore, Darmstadt, Germany), goat monoclonal anti-CD31 (~140 kDa; 1:500; Santa Cruz Biotechnologies, Santa Cruz, CA), mouse monoclonal anti-smooth muscle α-actin (~43 kDa; 1:5000; Sigma-Aldrich), rabbit polyclonal anti-collagen type I (~240 KDa pro-collagen;1:1000; Abcam, Cambridge, MA), rabbit polyclonal anti-prolyl 4-hydroxylase alpha polypeptide III (~75 KDa;1:500; EnoGene, New York, NY) and a mouse monoclonal anti-GAPDH (~37 kDa; 1:50,000; Ambion, Austin TX). Following overnight incubation at 4°C, the appropriate secondary antibody-conjugated to horseradish peroxidase (1:20,000, Jackson Immunoresearch, West Grove, PA) was added and bands visualized utilizing the ECL detection kit (Perkin Elmer). Films were scanned with Image J software^®^ and the target protein signal was depicted as arbitrary light units normalized to GAPDH protein.

### Immunofluorescence

Adult rat hearts were fixed in 2-methylbutane and cryosections of 14 μm were used for immunofluorescence, as previously described.[11] Cryosections were stained with the mouse monoclonal anti-nestin (1:400; EMD Millipore), rabbit polyclonal anti-smooth muscle α-actin (1:400; Abcam), rabbit polyclonal anti-collagen type I (1:400; Abcam), or goat monoclonal anti-CD31 (1:100; Santa Cruz Biotechnologies).[14] Adult and neonatal ventricular fibroblasts, rat microvascular and human coronary artery endothelial cells were plated on glass coverslips, fixed with 4% paraformaldehyde and stained with the mouse monoclonal anti-nestin (1:500; EMD Millipore), mouse monoclonal anti-human nestin (1:100; Santa Cruz Biotechnologies), mouse monoclonal anti-eNOS (1:50; BD Bioscience, Mississauga, ON), goat monoclonal anti-CD31 (1:100; Santa Cruz Biotechnologies, Santa Cruz, CA), rabbit polyclonal anti-prolyl 4-hydroxylase alpha polypeptide III (~75 KDa;1:100; EnoGene) or rabbit polyclonal anti-collagen type I (1:400; Abcam).[6] The nucleus was identified with To-PRO-3 (1.5 μM; ThermoFischer Scientific, Waltham, MA) or 4’,6’-diamidino-2-phenylindole (DAPI, Sigma-Aldrich; 1.5 μM).[6,14] Secondary antibodies used were a goat anti-mouse IgG conjugated to Alexa-555 (1:800; emission wavelength, 570 nm; Life Technologies, Carlsbad, CA), a goat anti-rabbit IgG conjugated to Alexa-488 (1:800; emission wavelength, 520 nm; Life Technologies), or a donkey anti-rabbit IgG conjugated to Alexa-647 (1:800; emission wavelength, 668 nm; Life Technologies). Immunofluorescence was visualized using a confocal LSM710 Zeiss microscope with the Zeiss LSM Image Browser. To determine the number of collagen type 1^(+)^- and collagen type I/nestin^(+)^-cells, interstitial cells expressing the mesenchymal protein were first identified and nestin co-expression subsequently assessed. The number of cells was normalized to the area (mm^2^) and at least three images of the left ventricle per rat were examined. Non-specific staining was determined following the addition of the conjugated secondary antibody alone.

### Statistics

Data were presented as the mean±S.E.M and (n) represented the number of rats, individual preparation of ventricular or endothelial cells used per experiment. Data was evaluated by a student’s unpaired t-test or a one-way ANOVA followed by Student-Newman-Keuls multiple comparisons post-hoc test (GraphPad InStat) and a value of *P*<0.05 was considered statistically significant.

## Results

### Left ventricular contractility and cardiovascular remodelling of suprarenal abdominal aorta constricted rats

**Fig 1:**
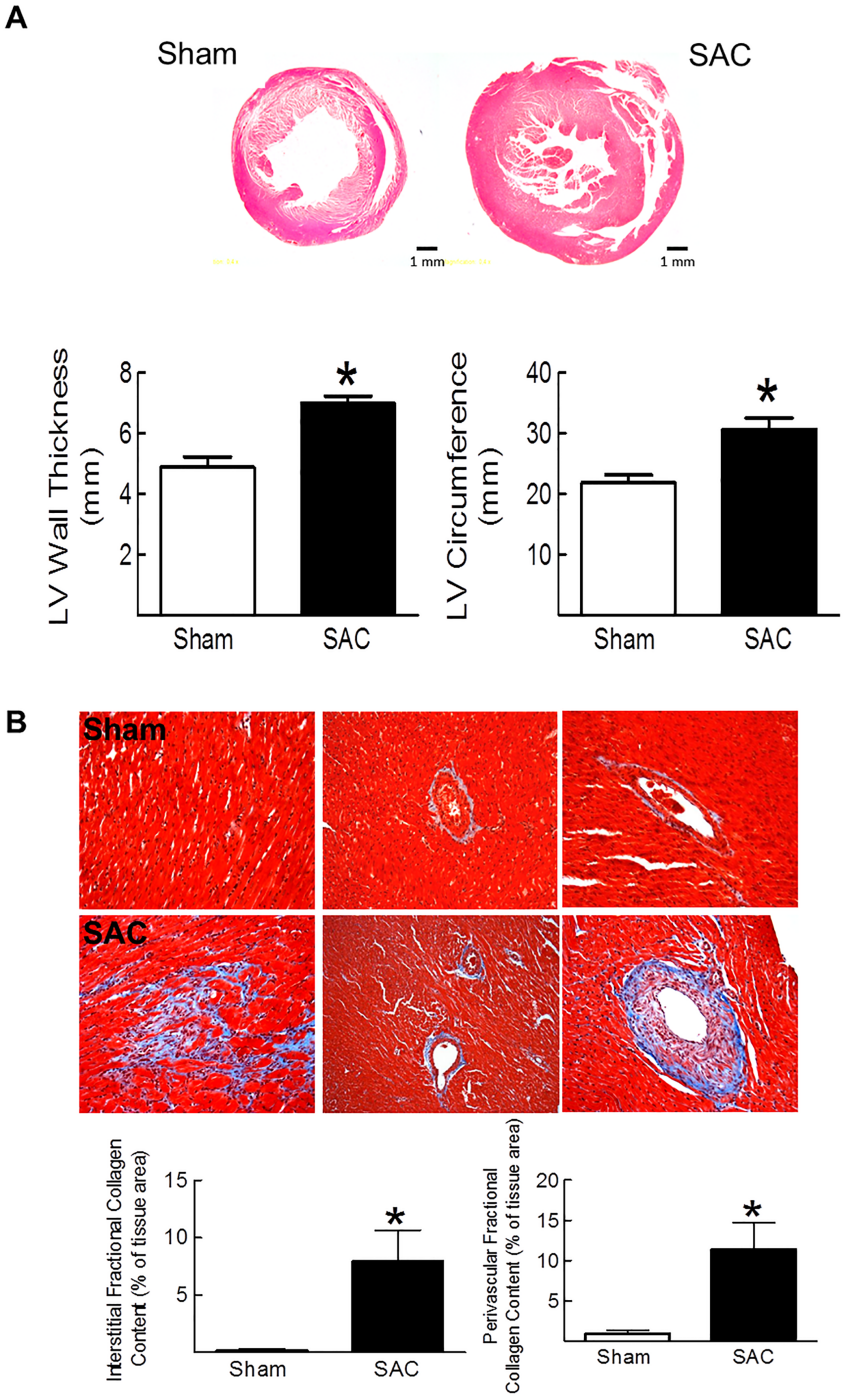
Pressure-overload induced concentric hypertrophy and reactive fibrosis. (**Panel A)** Left ventricular (LV) wall thickness, circumference, **(Panel B**) interstitial and perivascular fibrosis as determined by Masson Trichrome staining were significantly increased in suprarenal abdominal aorta constricted rats (SAC) versus sham rats. Analysis was performed on a subpopulation of SAC (n = 3) and sham (n = 4) rats and (*) denotes *p*<0.05 versus sham as determined by a student’s unpaired t-test.

Two weeks following suprarenal abdominal aorta constriction of adult male Sprague-Dawley rats, body weight was similar to sham rats whereas left and right ventricular weights were increased after a greater hemodynamic afterload was imposed (Table 1). Mean arterial pressure, left ventricular systolic pressure, the rate of left ventricular contraction and relaxation were significantly elevated in suprarenal abdominal aorta constricted (SAC) adults rats (Table 2). Cardiac remodelling after suprarenal abdominal aorta constriction was further characterized by an increase in left ventricular wall thickness and circumference as compared to sham rats (Fig 1A). Lastly, concentric remodelling of the left ventricle was associated with a reactive fibrotic response characterized by the extensive accumulation of collagen in the interstitium and perivascular region of numerous small and large calibre blood vessels (Fig 1B).

**Table 1:** Body and heart weight of sham and 2-week Suprarenal Abdominal Aorta Constricted (SAC) rats.

	BW (g)	Heart (g)	LV (mg)	RV (mg)
Sham (n = 8)	406±8	1.28±0.09	590±39	230±16
SAC (n = 8)	396±8	1.56±0.07*	801±49*	284±9*

BW indicates body weight; LV, left ventricle and RV, right ventricle. Data are presented as mean ± SEM, analyzed by a student’s unpaired t-test,

(*) represents *P*<0.05 versus sham and (n) number of rats examined.

**Table 2:** Hemodynamics of sham and 2-week Suprarenal Abdominal Aorta Constricted (SAC) rats.

	MAP (mmHg)	LVSP (mmHg)	LVEDP (mmHg)	+ dP/dT (mmHg/sec)	-dP/dT (mmHg/sec)
Sham (n = 7–8)	98±3	120±4	7±1	5843±168	4724±156
SAC (n = 6–8)	132±6*	193±20*	7±1	7616±421*	7747±810*

MAP indicates mean arterial pressure, LVSP, left ventricular systolic pressure, LVEDP, left ventricular end-diastolic pressure, +dp/dt, rate of contraction; -dp/dt rate of relaxation, LV, left ventricle, LR, right ventricle. Data are presented as mean ± SEM, analyzed by a student’s unpaired t-test.

(*) represents *P*<0.05 versus sham and (n) number of rats examined.

### Nestin protein levels were increased in the hypertrophied left ventricle of SAC rats and detected in cardiomyocytes, mesenchymal cells and interstitial CD31^(+)^-cells

**Fig 2:**
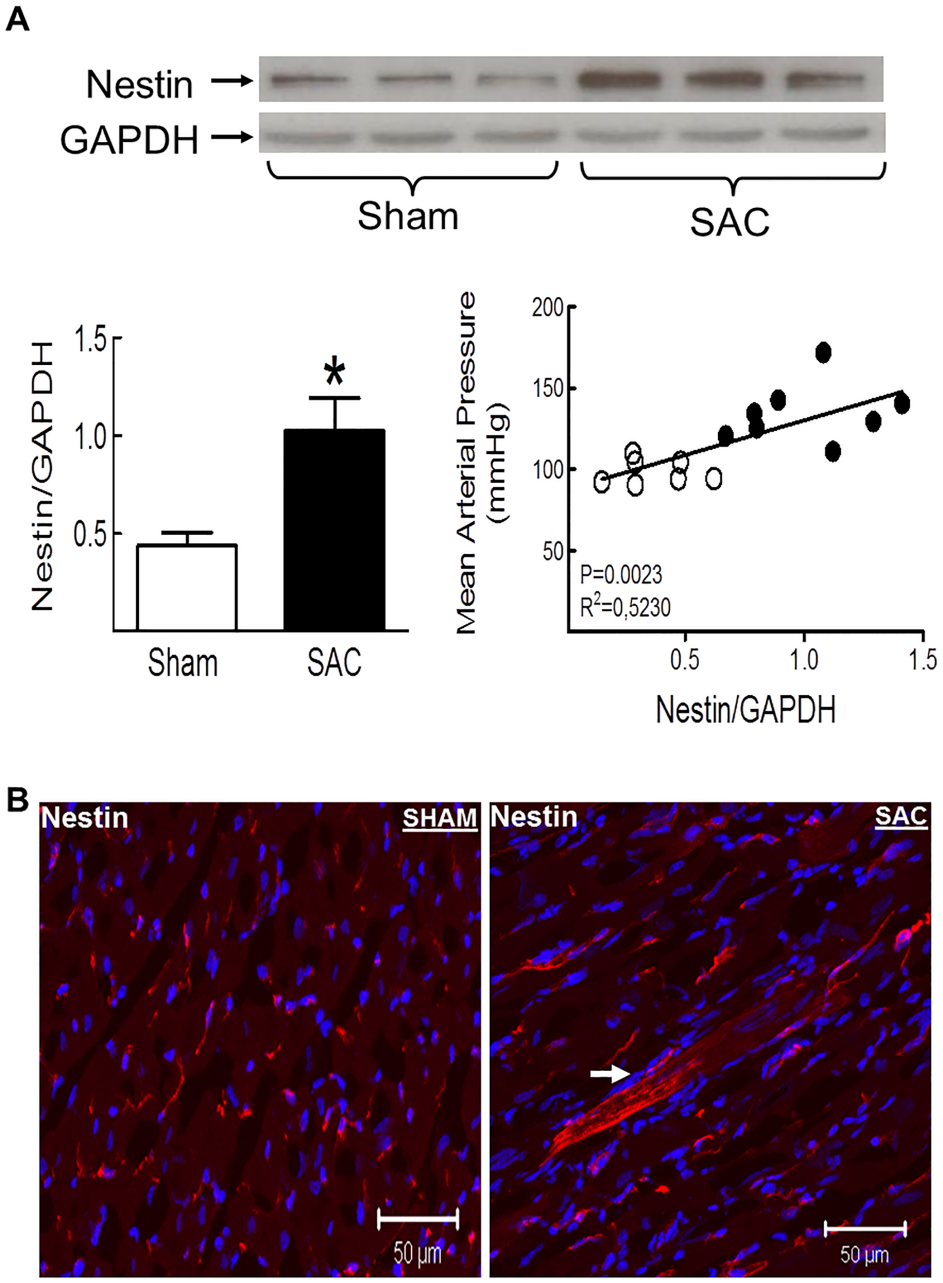
Nestin protein expression and appearance of nestin^(+)^-cardiomyocytes in the left ventricle of the pressure-overloaded rat heart. **(Panel A)** Nestin protein levels were significantly increased in the left ventricle of suprarenal abdominal aorta constricted (SAC) rats as compared to sham rats. Nestin protein expression in the left ventricle of sham (open circles) and SAC (filled circles) rats positively correlated with the rise of mean arterial pressure. Nestin protein expression was normalized to GAPDH, n = 7 sham rats, n = 8 banded rats and (*) denotes *P*<0.05 versus sham as determined by a student’s unpaired t-test. **(Panel B)** In the normal left ventricle, nestin immunoreactivity was observed exclusively in cells detected intercalated between cardiomyocytes. In the left ventricle of SAC rats, a paucity of cardiomyocytes (indicated by arrow) was associated with nestin immunoreactivity.

**Fig 3:**
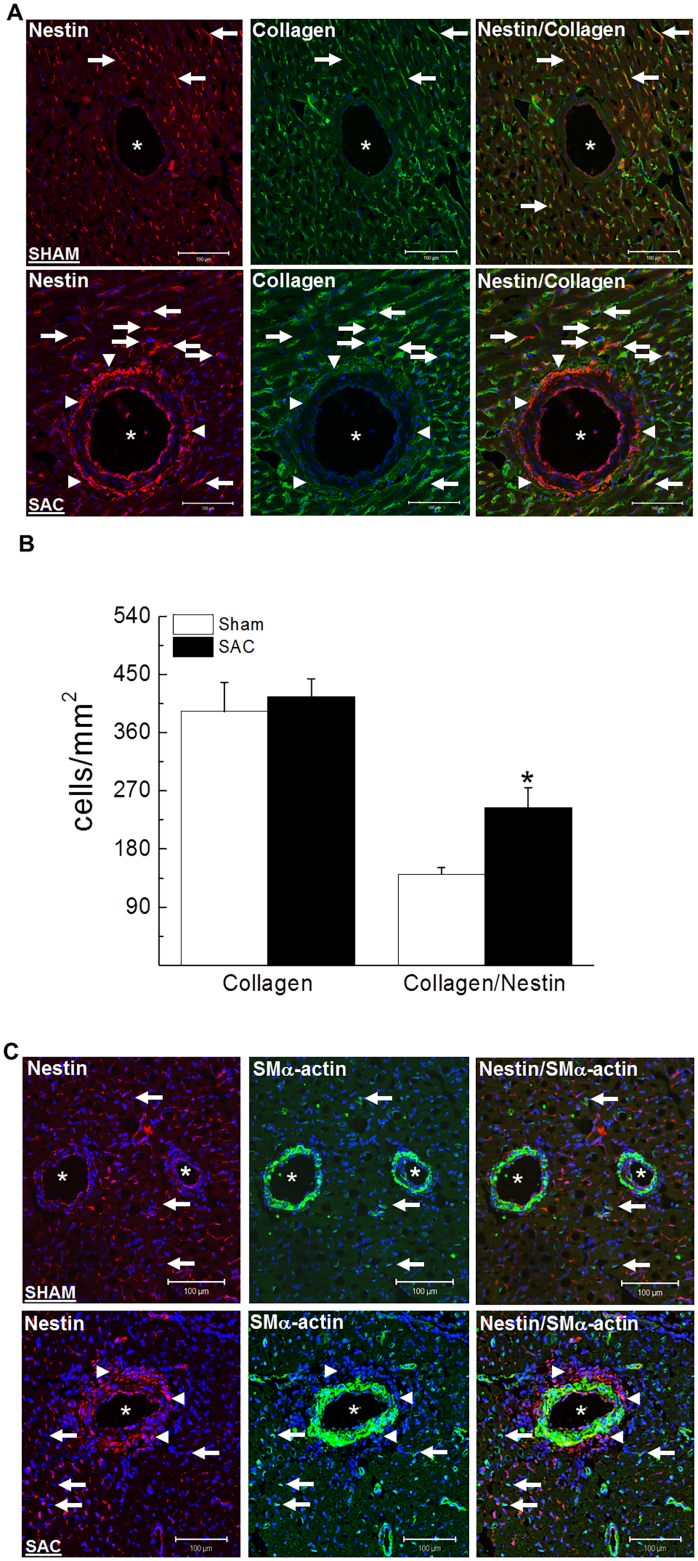
The phenotype of interstitial and perivascular cells. **(Panel A)** In the heart of sham and suprarenal abdominal aorta constricted rats (SAC), a subpopulation of interstitial and perivascular collagen type I^(+)^-cells co-expressed the intermediate filament protein nestin (indicated by arrow). Furthermore, in the pressure-overloaded heart, the selective accumulation of nestin^(+)^-cells (indicated by arrowhead) at the adventitial region of predominantly large caliber blood vessels lacked collagen type I staining. (**Panel B**) A semi-quantitative assessment revealed that the number of interstitial collagen type I^(+)^-cells (normalized to the area;mm^2^) was similar between the left ventricle of sham (n = 3) and suprarenal abdominal aorta constricted rats (SAC) (n = 3). By contrast, a significantly greater number of collagen type I^(+)^-cells co-expressing the intermediate filament protein nestin was observed in the heart of SAC rats as compared to sham rats. (*) denotes *p*<0.05 versus sham group as determined by a student’s unpaired t-test. (**Panel C**) Nestin staining expression was not detected in interstitial smooth muscle α-actin^(+)^-cells (indicated by arrow) of the sham and suprarenal abdominal aorta constricted rats (SAC). Smooth muscle α-actin staining was detected in vascular smooth muscle cells and a paucity co-expressed nestin in the heart of pressure-overloaded rats. Furthermore, the accumulation of nestin^(+)^-cells (indicated by arrowhead) detected exclusively in the adventitial region of predominantly large caliber blood vessels that lacked collagen type I staining were also not immunoreactive to smooth muscle α-actin. Blood vessels are indicated by an asterisk, the nucleus identified by To-PRO-3 (blue fluorescence) and n = 3–4 rats were examined in sham and pressure-overloaded groups.

**Fig 4:**
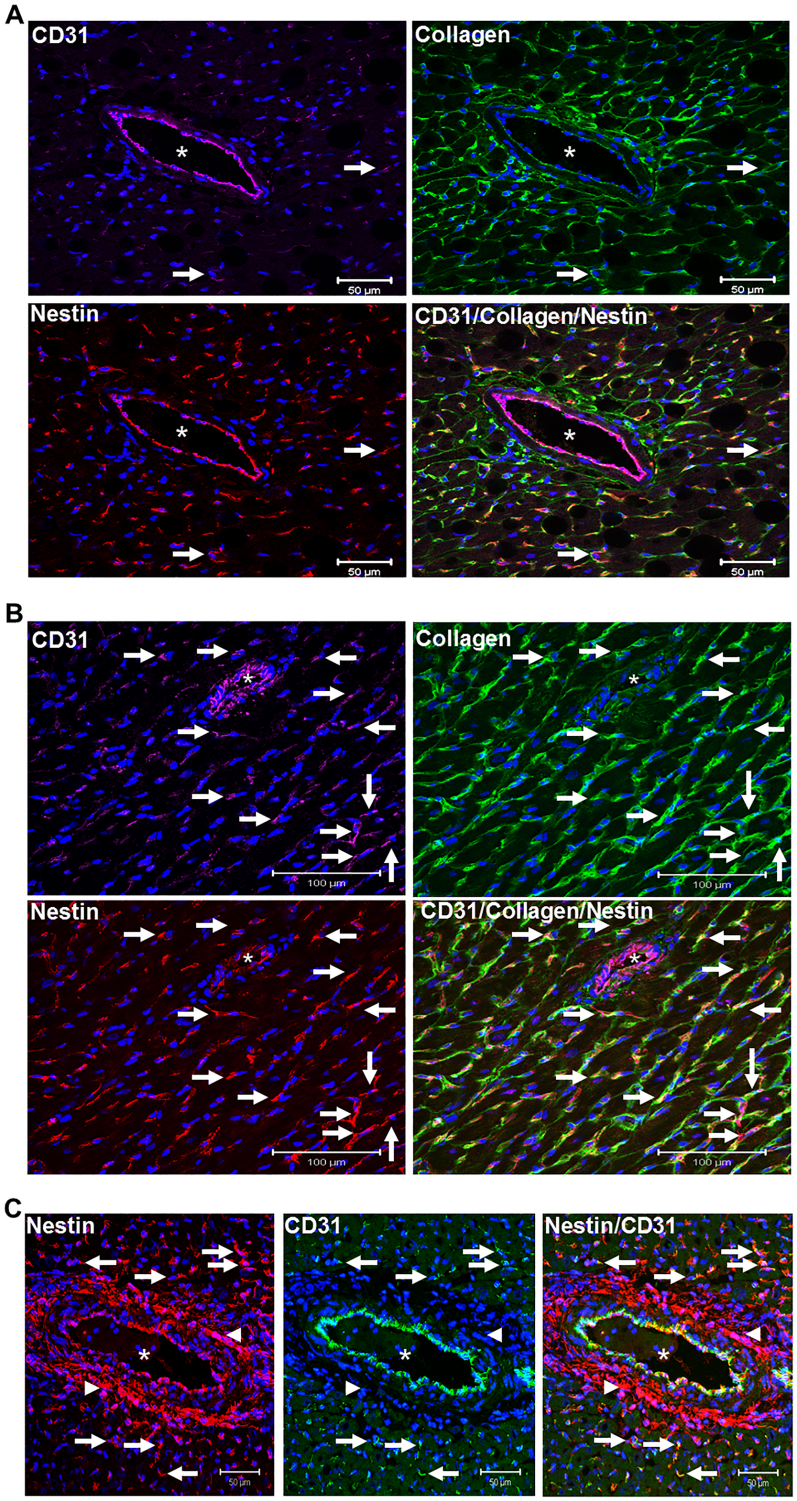
CD31 staining of endothelial and interstitial cells in the sham and hypertrophied left ventricle. (**Panel A**) In heart of sham rats, CD31 staining was detected in endothelial cells of blood vessels residing in the left ventricle. A paucity of CD31^(+)^-interstitial cells (indicated by arrow) were detected in close proximity to blood vessels and co-expressed nestin and collagen type I. (**Panels B & C)** CD31 staining of endothelial cells persisted in blood vessels residing in the left ventricle of suprarenal abdominal aorta constricted rats. A greater number of CD31^(+)^- interstitial cells (indicated by arrow) co-expressing nestin and collagen type I was observed in the left ventricle of suprarenal abdominal aorta constricted rats. (**Panel C**) The selective accumulation of nestin^(+)^-cells (indicated by arrowhead) at the adventitial region of predominantly large caliber blood vessels of suprarenal abdominal aorta constricted rats that lacked collagen type I staining were not immunoreactive to CD31. By contrast, in close proximity to the adventitial region, a subpopulation of interstitial CD31^(+)^-cells co-expressed nestin (indicated by arrow). Blood vessels are indicated by an asterisk, the nucleus identified by To-PRO-3 (blue fluorescence) and n = 3–4 rats were examined in sham and suprarenal abdominal aorta constricted groups.

Nestin protein levels were significantly increased in the hypertrophied/fibrotic left ventricle of SAC rats and the magnitude of expression positively correlated with the rise of mean arterial blood pressure (Fig 2A). Employing an immunofluorescence approach, a modest population of adult cardiomyocytes identified exclusively in the left ventricle of pressure-overloaded hearts expressed nestin (Fig 2B). In the left ventricle of sham and SAC rats, interstitial mesenchymal cells characterized by collagen type I immunoreactivity were detected and a subpopulation co-expressed the intermediate filament protein nestin (Fig 3A). A semi-quantitative assessment by immunofluorescence revealed that the number of collagen type I^(+)^-cells in the left ventricle of sham and SAC rats were similar (Fig 3B). However the number of collagen type I^(+)^-cells that co-expressed the intermediate filament protein nestin was significantly greater (69%↑) in the left ventricle of pressure-overloaded hearts versus sham hearts (Fig 3B). Furthermore, in stark contrast to sham rats, an important accumulation of nestin^(+)^-cells that lacked collagen type I immunoreactivity was identified at the adventitia of predominantly larger calibre blood vessels of SAC rats (Fig 3A). In sham and pressure-overloaded hearts, smooth muscle α-actin immunoreactivity was detected in interstitial and vascular smooth muscle cells (Fig 3C). Nestin co-expression was observed in a paucity of vascular smooth muscle cells identified in the hypertrophied left ventricle whereas the intermediate filament protein was not detected in interstitial smooth muscle α-actin^(+)^-cells (Fig 3C). Nestin immunoreactivity was also identified in CD31^(+)^-endothelial cells of blood vessels in the left ventricle of sham and SAC rats (Fig 4A and 4B). A modest number of interstitial cells expressing the endothelial marker CD31 were detected in close proximity to the vasculature of the normal left ventricle of sham rats and a subpopulation co-expressed nestin and collagen type I (Fig 4A). By contrast, a greater number of interstitial CD31^(+)^-immunoreactive cells co-expressing nestin and collagen type I was identified in the pressure-overloaded heart (Fig 4B). Collagen type I immunoreactivity was absent in CD31^(+)^-endothelial cells identified in the vasculature of sham and SAC rats (Fig 4A and 4B). Lastly, the accumulation of nestin^(+)^-cells at the adventitial region of predominantly larger calibre blood vessels of pressure-overloaded hearts that lacked collagen type I immunoreactivity likewise failed to co-express CD31 (Fig 4C).

### The exposure of ventricular fibroblasts to a panel of pro-fibrotic peptide growth factors increased expression of the intermediate filament protein nestin

**Fig 5:**
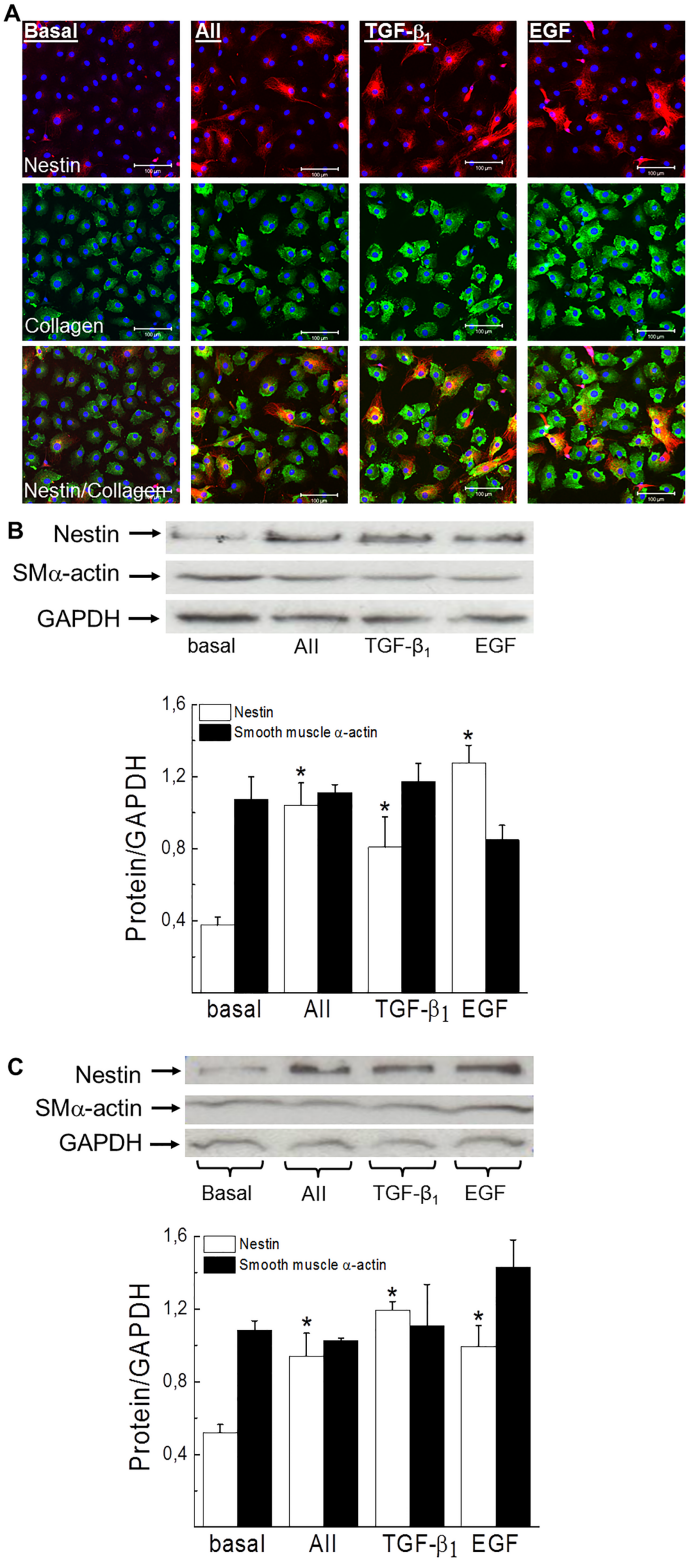
Nestin protein expression and DNA synthesis in adult ventricular fibroblasts. (**Panel A**) A paucity of collagen type I^(+)^-adult ventricular neonatal fibroblasts expressed nestin. A 24 hour exposure of adult ventricular fibroblasts to angiotensin II (AII; 1 μM), transforming growth factor-β_1_ (TGF-β_1_;5 ng/ml) or epidermal growth factor (EGF; 10 ng/ml) increased the appearance of fibroblasts expressing filamentous nestin. The nucleus was identified by To-PRO-3 (blue fluorescence) and n = 3 normal adult rats were examined. The exposure of ventricular fibroblasts isolated from the normal (**Panel B**) and hypertrophied heart (**Panel C**) to angiotensin II, transforming growth factor-β_1_ or epidermal growth factor for 24 hrs significantly increased nestin protein expression whereas smooth muscle α-actin levels remained unchanged. Protein expression was normalized to GAPDH, n = 3–4 independent experiments derived from normal and hypertrophied hearts and (*) denotes *p*<0.05 versus cells in an untreated basal state as determined by a student’s unpaired t-test.

**Fig 6:**
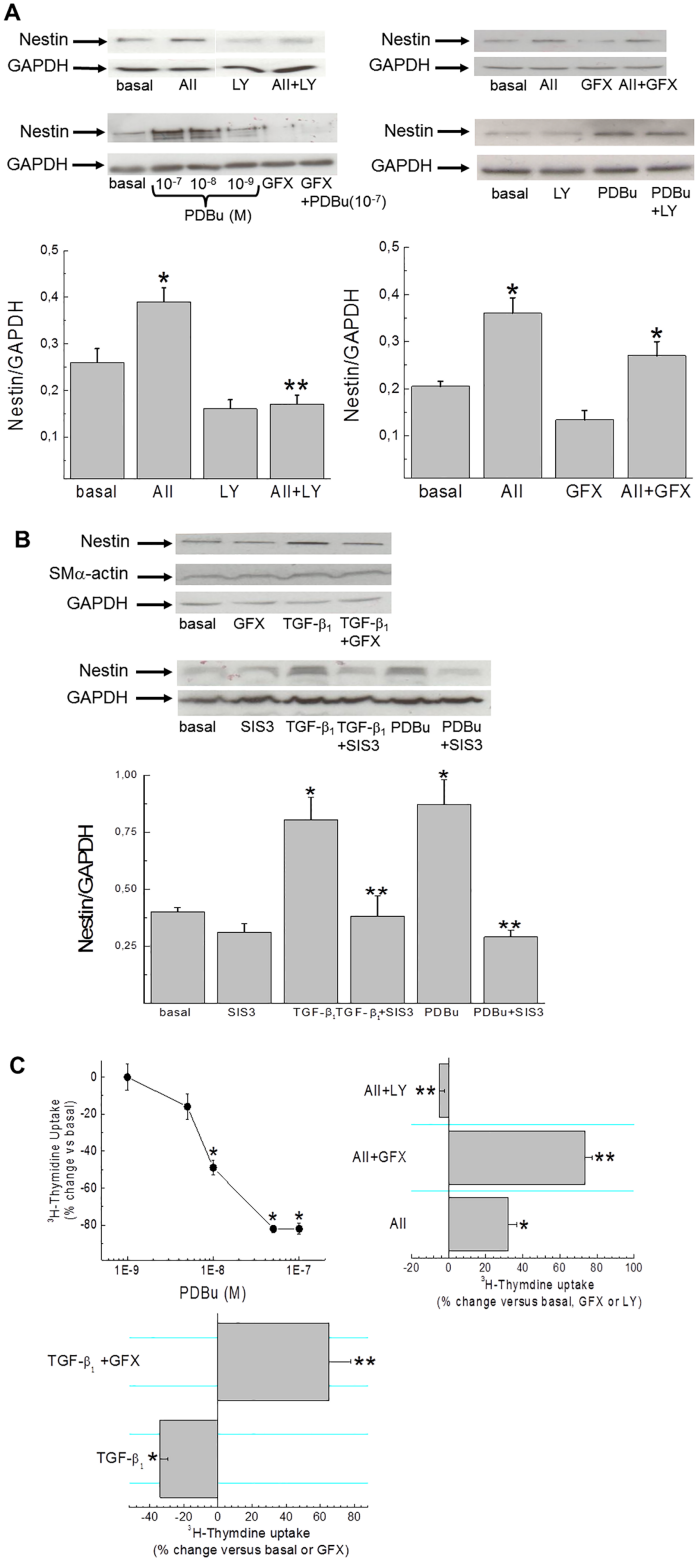
Signalling events coupled to nestin protein expression and DNA synthesis in neonatal rat ventricular fibroblasts. (**Panel A**) Angiotensin II (AII; 1 μM) stimulation of neonatal rat ventricular fibroblasts for 24 hours increased nestin protein levels and LY294002 (LY; 10 μM; n = 3) pre-treatment inhibited the response whereas pre-treatment with GF109203X (GFX; 1 μM; n = 3) was without effect. Phorbol 12,13-dibutyrate (PDBu; 100nM) stimulation for 24 hours led to a dose-dependent increase of nestin protein levels and GF109203X (n = 3) pre-treatment inhibited the response whereas LY294002 (n = 2) was without effect. Protein expression was normalized to GAPDH, (*) denotes *p*<0.05 versus cells in an untreated basal state or GF109203X/LY294002 treatment alone and (**) denotes *p*<0.05 versus AII as determined by a one-way ANOVA. (**Panel B**) Transforming growth factor-β_1_ (TGF-β_1_; 5 ng/ml) stimulation for 24 hours increased nestin protein levels and pre-treatment with GF109203X (n = 2) or SIS3 (5 μM;n = 3) inhibited the response. The inhibitor SIS3 (n = 4) likewise suppressed phorbol 12,13-dibutyrate mediated nestin expression. Protein expression was normalized to GAPDH, (*) denotes *p*<0.05 versus cells in an untreated basal state or SIS3 treatment alone and (**) *p*<0.05 versus TGF-β_1_ or PDBu as determined by a one-way ANOVA. (**Panel C**) PDBu stimulation of neonatal rat ventricular fibroblasts for 24 hours led to dose dependent suppression of ^3^H-thymidine uptake. Angiotensin II (AII; 1 μM) stimulation of neonatal ventricular fibroblasts for 24 hrs increased ^3^H-thymidine uptake and pre-treatment with LY294002 (LY;10 μM) inhibited the response whereas GF109203X (GFX;1 μM) pre-treatment potentiated the increase in DNA synthesis. TGF-β_1_ mediated inhibition of ^3^H-thymidine uptake was reversed and DNA synthesis was further increased after the pre-treatment with GF109203X. N = 4–5 independent preparations of neonatal rat ventricular fibroblasts, (*) denotes *p*<0.05 versus cells in an untreated basal state and (**) p<0.05 versus AII or TGF-β_1_ as determined by a student’s unpaired t-test.

Ventricular fibroblasts isolated from the heart of normal adult rats were characterized by collagen type I immunoreactivity and a modest population expressed filamentous nestin (Fig 5A). A 24 hour exposure of normal adult collagen type I^(+)^-fibroblasts to the pro-fibrotic peptide growth factor angiotensin II (1 μM), transforming growth factor-β_1_ (5 ng/ml) or epidermal growth factor (10 ng/ml) increased the appearance of fibroblasts expressing filamentous nestin (Fig 5A). Consistent with the latter data, nestin protein levels were significantly elevated whereas smooth muscle α-actin protein expression in normal adult ventricular fibroblasts was unchanged in response to a panel of pro-fibrotic peptide growth factors (Fig 5B). An analogous pattern of nestin and smooth muscle α-actin expression was observed in ventricular fibroblasts isolated from the hypertrophied heart of SAC rats (Fig 5C) and the heart of neonatal rats (Fig 6A and 6B) in response to a panel of pro-fibrotic peptide growth factors. The latter data supported the use of neonatal ventricular fibroblasts to examine the signalling events coupled to nestin protein expression. Pre-treatment with the protein kinase C inhibitor GF109203X (1 μM) failed to inhibit AII-mediated nestin expression whereas the PI3-K inhibitor LY294002 (10 μM) abrogated the response (Fig 6A).[18,19] By contrast, GF109203X pre-treatment inhibited TGF-β_1_ mediated nestin expression (basal = 0,35±0,03, TGF-β_1_ = 0,62±0,02, TGF-β_1_+GF109203X = 0,38±0,08;n = 2) (Fig 6B). Furthermore, pre-treatment with the selective inhibitor of SMAD3 phosphorylation SIS3 (5 μM) suppressed nestin upregulation in response to TGF-β_1_ (Fig 6B).[20] The 24 hour exposure of neonatal ventricular fibroblasts to the PKC activator phorbol 12,13 dibutyrate (PDBu; 100 nM) increased nestin protein levels and the response was inhibited by GF109203X and SIS3 (Fig 6A and 6B). By contrast, LY294002 did not inhibit nestin protein expression following exposure to PDBu (basal = 0,27±0,02, PDBu = 0,68±0,11, PDBu+LY294002 = 0,60±0,02;n = 2) (Fig 6A). Despite nestin upregulation, TGF-β_1_ and PDBu treatment for 24 hrs inhibited ^3^H-thymidine uptake in neonatal ventricular fibroblasts (Fig 6C). TGF-β_1_ mediated inhibition of DNA synthesis was reversed and potentiated by GF109203X (Fig 6C). The 24 hour exposure of neonatal ventricular fibroblasts to AII increased DNA synthesis and LY294002 pre-treatment inhibited the response whereas GF109203X potentiated ^3^H-thymidine uptake (Fig 6C). A previous study reported that neonatal rat ventricular fibroblasts expressed PKCα and GF109203X inhibition of this isoform may in part explain the potentiation of AII- and TGF-β_1_ mediated DNA synthesis.[21] Indeed, the 24 hr treatment of neonatal ventricular fibroblasts with the cell permeable N-myristolated PKC (20–28) inhibitor (10 μM) that selectively targeted the PKCα/β-isoforms potentiated the basal uptake of ^3^H-thymidine (77±12%↑ versus basal; n = 4).

### Rat microvascular endothelial cells possess an intrinsic mesenchymal phenotype and exposure to pro-fibrotic peptide growth factors induced nestin expression

**Fig 7:**
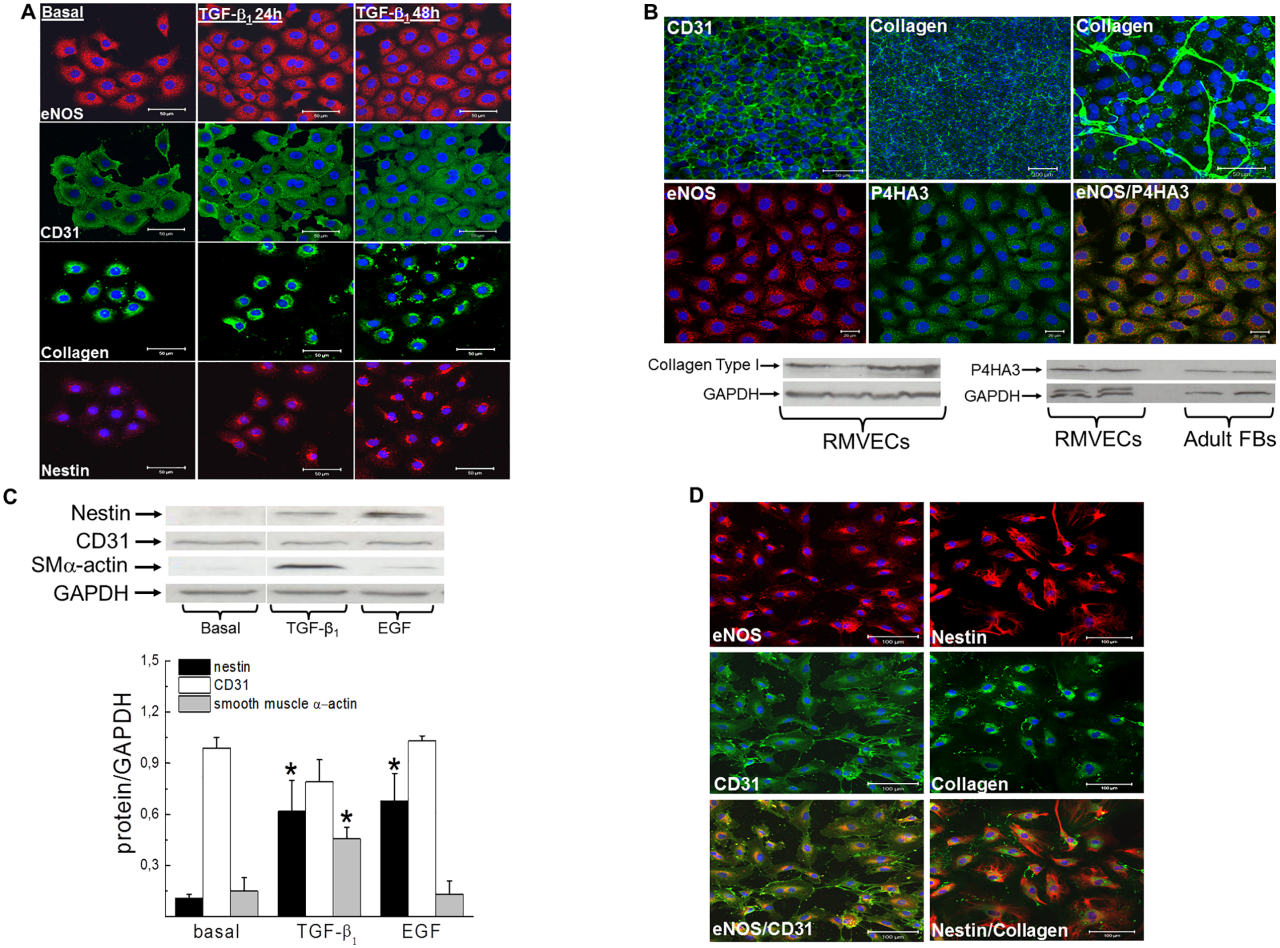
Endothelial cells expressed mesenchymal proteins and pro-fibrotic peptide growth factors induced nestin. **(Panel A)** Collagen type I immunoreactivity was detected in eNOS/CD31^(+)^-rat microvascular endothelial cells whereas nestin staining was modest. Transforming growth factor-β_1_ (TGF-β_1_;5 ng/ml) treatment increased nestin expression whereas eNOS and CD31 staining remained intact. (**Panel B**) Rat microvascular endothelial cells (RMVECs) expressed collagen type I and prolyl 4 hydroxylase A3 (P4HA3) and P4HA3 was also detected in adult ventricular fibroblasts (FBs). In eNOS^(+)^-RMVECS, a punctate pattern of P4HA3 staining was observed. Upon confluency, CD31 translocated to the plasma membrane and identifiable junctions were evident between RMVECs. Furthermore, collagen type I protein was secreted leading to the formation of a fibrillary network. (**Panel C**) A 24 hour exposure to TGF-β_1_ (5 ng/ml) and epidermal growth factor (EGF;10 ng/ml) significantly upregulated nestin protein levels whereas CD31 protein expression was unchanged. Furthermore, TGF-β_1_ increased smooth muscle α-actin protein levels in rat microvascular endothelial cells. Protein expression was normalized to GAPDH, n = 3–4 and (*) denotes *p*<0.05 versus cells in an untreated basal state as determined by a student’s unpaired t-test. (**Panel D**) Collagen type I and filamentous nestin immunoreactivity were detected in eNOS/CD31^(+)^-human coronary artery endothelial cells.

The accumulation of interstitial CD31^(+)^-cells co-expressing nestin and collagen type I in the hypertrophied left ventricle of SAC rats provided the impetus to examine whether this population may represent displaced endothelial cells. As expected, CD31 and eNOS immunoreactivity was detected in rat cardiac microvascular endothelial cells (Fig 7A). In sub-confluent conditions, CD31 and eNOS staining was observed in the cytoplasm and eNOS immunoreactivity was further characterized by a punctate pattern (Fig 7A). In confluent rat cardiac microvascular endothelial cells, CD31 translocated to the plasma membrane (Fig 7B). In the absence of endothelial-mesenchymal transition, CD31/eNOS^(+)^-rat cardiac microvascular endothelial cells expressed collagen type 1 and upon confluency the extracellular matrix protein was secreted leading to the formation of a fibrillary network (Fig 7A and 7B). To reaffirm a fibrotic phenotype, the enzyme implicated in collagen synthesis prolyl 4-hydroxylase A3 was detected by Western blot and an immunofluorescence approach revealed a punctate pattern of staining (Fig 7B). [22] In contrast to collagen type I, nestin protein levels were modest or undetectable in rat cardiac microvascular endothelial cells (Fig 7A). A 24 hr exposure to transforming growth factor-β_1_ (5 ng/ml) or epidermal growth factor (10 ng/ml) significantly upregulated nestin protein levels (Fig 7C). However, the intermediate filament protein was not organized in a filamentous pattern but predominantly localized to the Golgi region following transforming growth factor-β_1_ stimulation (Fig 7A). Furthermore, exposure of rat cardiac microvascular endothelial cells to transforming growth factor-β_1_ induced smooth muscle α-actin expression, whereas epidermal growth factor had no effect (Fig 7C). Despite nestin and smooth muscle α-actin upregulation in response to pro-fibrotic peptide growth factors, CD31 and eNOS expression persisted in rat cardiac microvascular endothelial cells (Fig 7A and 7C). Lastly, the extracellular matrix protein collagen type I was detected in CD31/eNOS^(+)^-human coronary artery endothelial cells and in contrast to rat microvascular endothelial cells expressed filamentous nestin (Fig 7D).

## Discussion

Work from our lab and others have reported the selective appearance of a modest population of nestin^(+)^-cardiomyocytes at the peri-infarct/infarct region following ischemic damage.[23,24] In the present study, a paucity of nestin^(+)^-cardiomyocytes was detected in the hypertrophied heart after suprarenal abdominal aorta constriction of adult male Sprague-Dawley rats. However, hypertrophy alone was not sufficient to induce nestin expression in neonatal and adult cardiomyocytes.[11,23] However, concentric remodeling of the heart was associated with ischemic injury and may represent the incipient event promoting in part the subsequent reactive fibrotic response.[25] Thus, the appearance of nestin^(+)^-cardiomyocytes in the pressure-overloaded hypertrophied rat heart may be secondary to ectopic ischemic injury.

The increased synthesis and deposition of collagen by activated ventricular fibroblasts contributed to the establishment of interstitial and perivascular fibrosis in the heart of SAC rats. However, a marker identifying the activated state of ventricular fibroblasts in the setting of reactive fibrosis remains equivocal. Previous studies have reported that smooth muscle α-actin expression characterized activated fibroblasts following tissue injury.[1,2] Nonetheless, the latter paradigm was challenged by several recent studies demonstrating that the majority of collagen type I^(+)^-mesenchymal cells identified in the interstitial and perivascular regions of the fibrotic pressure-overloaded mouse heart and in the fibrotic rat lungs secondary to hypobaric hypoxia lacked smooth muscle α-actin immunoreactivity.[3,11] Work from our lab revealed that the intermediate filament protein nestin was highly expressed in neonatal rat ventricular fibroblasts, significantly downregulated in adult rat ventricular fibroblasts and recapitulated in scar myofibroblasts during the reparative fibrotic response following myocardial infarction.[6] Nestin was also detected in mesenchymal cells identified in the fibrotic lung and kidney and the accumulation of interstitial collagen following injury of the rat and human kidney positively correlated with the density of nestin^(+)^-interstitial cells.[11–13] In the present study, collagen^(+)^-mesenchymal cells co-expressing nestin were detected in the sham and hypertrophied/fibrotic heart supporting the premise that this population may represent in part resident ventricular fibroblasts. Moreover, in the heart of SAC rats, the number of collagen type I^(+)^-mesenchymal cells that co-expressed nestin was significantly greater than that observed in the heart of sham rats. By contrast, nestin immunoreactivity was not detected in interstitial smooth muscle α-actin^(+)^-cells in the left ventricle of normal and SAC rats. Consistent with these findings, the exposure of collagen type I^(+)^-ventricular fibroblasts isolated from the normal and pressure-overloaded heart to putative pro-fibrotic peptide growth factors, AII, TGF-β_1_ and EGF induced nestin expression and increased the number of fibroblasts displaying a filamentous phenotype, whereas smooth muscle α-actin protein levels remained unchanged. These data support the premise that the greater number of collagen type I^(+)^-mesenchymal cells that co-expressed nestin contributed in part to the observed increase in protein levels of the intermediate filament protein in the heart of SAC rats. Furthermore, nestin rather than smooth muscle α-actin expression in adult ventricular fibroblasts may represent a bona fide marker of an activated phenotype during the progression of reactive fibrosis in the left ventricle of suprarenal abdominal aorta constricted rats.

In stark contrast to the normal heart, an important accumulation of nestin^(+)^-cells that lacked collagen type I and smooth muscle α-actin immunoreactivity was identified in the adventitial region of predominantly large caliber blood vessels in the heart of SAC rats. The cellular source of adventitial nestin^(+)^-cells and potential contribution to perivascular fibrosis remains presently unresolved. It is tempting to speculate that neural crest-derived cardiac resident nestin^(+)^-cells may have migrated to the vasculature and contributed to the perivascular fibrotic response via the synthesis and secretion of pro-fibrotic peptide growth factors.[26] Alternatively, a recent study by Ieronimakis and colleagues revealed that the perivascular fibrotic response in the heart of Duchenne muscular dystrophic mice was associated with the selective accumulation of Sca1^(+)^-cells in the adventitial region that robustly expressed TGF-β_1_ and were not immunoreactive for smooth muscle α-actin.[27] Thus, regardless the identity and potential contribution to the perivascular fibrotic response, the adventitial accumulation of nestin^(+)^-cells represents a novel phenotype of large caliber blood vessel remodeling secondary to pressure-overload.

Work from our lab has previously reported that β-adrenergic and AII stimulation of neonatal rat ventricular fibroblasts increased PI3-K activity and putative PI3-K inhibitors LY294002 and wortmannin inhibited the response.[18] Furthermore, the exposure of neonatal rat ventricular fibroblasts to AII led to ERK1/2 phosphorylation via recruitment of PKC- and calcium-dependent pathways. [21] In the present study, AII stimulation of neonatal rat ventricular fibroblasts increased ^3^H-thymidine uptake and the response was abrogated by the PI3-K inhibitor LY294002. These data were consistent with previous studies revealing a role of the PI3-K pathway in AII stimulated growth of ventricular fibroblasts.[18] By contrast, pre-treatment with the PKC inhibitor GF109203X that predominantly targeted the classical isoforms of PKC [19], potentiated AII stimulated DNA synthesis. A previous study reported that PKCα was the only classical isoform (e.g. DAG & Ca^2+^-dependent) expressed in neonatal rat ventricular fibroblasts and in some forms of cancer PKCα exerted a tumour suppressive action.[21,28] Consistent with the observed potentiation of the AII response following GF109203X pre-treatment, the exposure of neonatal ventricular fibroblasts to the cell permeable N-myristolated PKC (20–28) inhibitor that selectively targeted the PKCα/β-isoforms significantly increased the basal uptake of ^3^H-thymidine whereas treatment with phorbol 12,13-dibuytrate suppressed DNA synthesis. Despite the concomitant recruitment of PI3-K and PKC-dependent pathways, AII mediated upregulation of nestin protein levels was selectively abrogated by the PI3-K inhibitor LY294002. The latter data supported previous findings of an important interaction between the PI3-K signaling pathway, nestin and cell proliferation [29,30] and further suggested that AII recruitment of a PKC-dependent pathway may represent a negative feedback mechanism limiting the cell cycle re-entry of ventricular fibroblasts.

TGF-β_1_ and phorbol 12,13-dibuytrate treatment of neonatal rat ventricular fibroblasts significantly inhibited ^3^H-thymidine uptake and concomitantly upregulated nestin protein levels. These data support the premise that the biological actions of TGF-β may have occurred in part via a PKC-dependent pathway.[31–34] Indeed, the suppressive action of TGF-β_1_ on DNA synthesis following stimulation of neonatal rat ventricular fibroblasts was reversed by the PKC inhibitor GF109203X and a further increase in ^3^H-thymidine uptake was observed. These observations were in part analogous to the TGF-β_1_ paradox identified during cancer progression. TGF-β_1_ was reported to act as a tumor suppressor during the early stages whereas cell proliferation and induction of a metastatic phenotype was prevalent during the later stages.[35] Moreover, in some but not all forms of cancer, PKCα protein expression was downregulated and in PKCα knockout mice spontaneous intestinal tumors were identified.[28] Thus, the TGF-β_1_ paradox identified during cancer progression may apparently extend to neonatal rat ventricular fibroblasts. GF109203X pre-treatment also inhibited TGF-β_1_ mediated upregulation of nestin protein levels and the selective inhibitor of SMAD3 phosphorylation SIS3 abrogated TGF-β_1_ and phorbol 12,13-dibuytrate induced expression of the intermediate filament protein.[21] These findings were consistent with previous studies demonstrating that PKC-dependent pathways acting upstream of SMAD-signaling events contributed in part to the various biological actions of TGF-β_1_.[31,32,34]

The reactive fibrotic response in the damaged kidney and lungs was associated with the appearance of interstitial cells expressing the endothelial cell markers CD31 or CD34 and a subpopulation co-expressed nestin.[11,12,36] Furthermore, interstitial CD31^(+)^-cells identified in the fibrotic lungs secondary to hypobaric hypoxia in the rat or patients with systemic sclerosis co-expressed collagen type I.[11,36] By contrast, collagen type I immunoreactivity was absent in CD31^(+)^-endothelial cells identified in the lung vasculature.[11] In the present study, a modest number of CD31^(+)^-interstitial cells detected in the sham rat heart co-expressed collagen type I and nestin and the preponderance were identified in close proximity to blood vessels. An analogous paradigm was observed in the hypertrophied/fibrotic left ventricle and the number of CD31/nestin/collagen type I^(+)^-interstitial cells was apparently greater. As reported in the lungs, CD31/nestin^(+)^-endothelial cells in the vasculature of the normal and pressure-overloaded rat heart did not stain for collagen type I.[11] These data suggest that endothelial cells may have been displaced during vascular remodeling of the hypertrophied/fibrotic left ventricle and co-expression of nestin and collagen type I reflects the initial stage of transformation to a mesenchymal phenotype. However, as previously discussed, three independent studies have reported that the reactive fibrotic response of the pressure-overloaded mouse heart was not secondary to epithelial- or endothelial-mesenchymal transformation but selectively attributed to resident fibroblasts.[3–5] Therefore, how do we reconcile the appearance of interstitial CD31^(+)^-interstitial cells co-expressing nestin and collagen type I in the pressure-overload heart in the absence of endothelial-mesenchymal transformation. Previous studies have reported a collagen-producing phenotype of endothelial cells and collagen type I was identified as a major isoform synthesized and secreted by corneal endothelial cells.[37–39] Furthermore, in two distinct models of hepatic fibrosis, collagen type I mRNA expression was 8–15 fold greater in endothelial cells as compared to normal hepatocytes.[39] In the present study, rat cardiac microvascular endothelial cells characterized by eNOS and CD31 staining expressed prolyl 4-hydroxylase A3, synthesized collagen type 1 and upon confluency the extracellular matrix protein was secreted and formation of a fibrillary network was observed. Furthermore, collagen type I immunoreactivity was identified in human coronary artery endothelial cells. By contrast, nestin expression in rat cardiac microvascular endothelial cells was modest or undetectable. Following TGF-β_1_ or EGF stimulation, nestin protein levels were increased whereas CD31 expression remained unchanged. In untreated human coronary artery endothelial cells, filamentous nestin staining was detected. The established phenotypic heterogeneity of endothelial cells which was in part dependent of the blood vessel calibre may partially explain the disparate pattern of nestin expression observed in coronary artery versus microvascular endothelial cells.[40] Collectively, these data suggest that human coronary artery and cardiac microvascular CD31^(+)^-endothelial cells apparently possess the intrinsic capacity to synthesize and secrete collagen type I in the absence of endothelial-mesenchymal transformation and putative pro-fibrotic peptide growth factors may play a seminal role inducing nestin expression. Based on the latter data, the significantly greater number of interstitial collagen type I/nestin^(+)^-cells identified in the hypertrophied left ventricle may be attributed in part to the increased appearance of displaced CD31^(+)^-endothelial cells displaying an unmasked mesenchymal phenotype. It is tempting to speculate that interstitial CD31^(+)^-cells may have contributed in part to the reactive fibrotic response of the left ventricle of SAC rats.

The present study has demonstrated that nestin upregulation in the hypertrophied/fibrotic left ventricle secondary to pressure-overload was attributed in part to the increased number of interstitial collagen type I cells co-expressing the intermediate filament protein. Indeed, the exposure of collagen type I^(+)^-ventricular fibroblasts to a panel of pro-fibrotic peptide growth factors induced nestin expression and may represent a marker of the activated phenotype during reactive fibrosis. Furthermore, a subpopulation of nestin^(+)^-cells was detected in the adventitial region of predominantly large calibre blood vessels in the pressure-overloaded heart, albeit their identity and role in the perivascular fibrotic response remains presently unresolved. The increased appearance of collagen type I/nestin/CD31^(+)^-cells in close proximity to blood vessels in the pressure-overloaded heart may represent displaced microvascular or coronary artery endothelial cells. The latter data was consistent with previous studies reporting the greater appearance of interstitial cells co-expressing endothelial and mesenchymal markers in the kidney and lung following injury.[11,12,36] Thus, collagen type 1 and prolyl 4-hydroxylase A3 expression in cardiac endothelial cells suggests that lineage tracing via these mesenchymal proteins may not exclusively identify an interstitial population of ventricular fibroblasts during reactive and reparative fibrosis.

## Supporting information

S1 File ARRIVE guidelines checklist. (PDF)
